# Policy frameworks for promoting public hospital development in major economies: implications for China

**DOI:** 10.3389/fpubh.2026.1774539

**Published:** 2026-02-23

**Authors:** Yuetian Shu, Zhiguang Li, Ruijin Xie

**Affiliations:** 1 School of Health Economics and Management, Anhui University of Chinese Medicine, Hefei, Anhui, China; 2 Nanjing Hospital of Chinese Medicine, Nanjing, Jiangsu, China; 3 Anhui Provincial Key Laboratory of Data Science and Innovative Development of Traditional Chinese Medicine, Hefei, Anhui, China

**Keywords:** China healthcare reform, compensation mechanism, international comparison, policy framework, public hospitals

## Abstract

**Background:**

As the core providers of medical and health services, public hospitals play an irreplaceable role in ensuring basic medical care, safeguarding health equity, and fulfilling social responsibilities. Their operational efficiency and institutional design not only influence the national health status but also relate to the stability and development of the entire healthcare system. Therefore, continuously optimizing support policies and management mechanisms for public hospitals has become a crucial issue for governments worldwide in advancing healthcare system reforms.

**Methods:**

This paper systematically reviews the policy trajectories supporting the development of public hospitals in five major global economies, namely the United States, the United Kingdom, Germany, Japan, and Singapore. Primary sources included government legislation and policy documents, official statistical publications issued by health authorities and insurance agencies, and peer-reviewed journal articles indexed in Web of Science, PubMed, and Scopus. The analysis focused on key institutional dimensions, including financing arrangements, governance structures, payment systems, and performance evaluation mechanisms. Through cross-country comparisons and longitudinal historical analysis, it reveals the commonalities and differences among various policy models.

**Results:**

The study finds that in the United States, support policies for public hospitals are primarily market-driven, supplemented by public programs. Public health insurance programs such as Medicare and Medicaid provide funding for public hospitals, and market-oriented management models are relied upon to enhance operational efficiency. The United Kingdom adopts the National Health Service system, which is based on tax financing and constructs a public hospital system that combines government leadership, internal marketization, and performance governance. Germany ensures the financial stability and operational efficiency of public hospitals through a dual governance structure of social health insurance and government capital investment. Under the universal health insurance system in Japan, a refined payment system and a local government responsibility-sharing mechanism drive public hospitals toward efficiency and equity. Singapore centers its approach on government subsidies and the 3M health insurance system, combined with group management of public hospitals, achieving equitable access and efficient operation of healthcare services. In China, the development of public hospitals follows a clear path of government leadership, with the basic medical insurance system serving as a solid guarantee. A diversified compensation mechanism has been established to ensure their stable operation. In terms of management, active efforts are made to advance the reform of separating management from operation while adhering to the core principle of public welfare orientation.

**Conclusions:**

There are differences among countries in the selection of policy tools and practical effects to support the development of public hospitals, but all demonstrate an ongoing balance between ensuring the supply of basic medical services, enhancing the efficiency of the service system, and addressing structural challenges. China should draw on international experience, taking into account its national conditions, to further strengthen the central government's coordinating role, clarify the boundaries of financial investment, optimize health insurance payment and price formation mechanisms, promote the reform of public hospitals toward legal person status and group management, accelerate digital transformation and the implementation of a tiered diagnosis and treatment system, and build an efficient, equitable, and sustainable development model for public hospitals.

## 1 Introduction

Public hospitals play a central role in national health systems by providing essential medical services, ensuring health equity, and safeguarding public health security. Their performance and sustainability are closely linked to broader policy environments, including financing mechanisms, governance structures, and healthcare delivery models. Understanding how different countries design and implement policy frameworks to support public hospitals is therefore critical for improving health system effectiveness and resilience.

Existing studies have examined selected aspects of public hospital reform, including the cost-containment effects of diagnosis-related group payment systems, governance changes associated with outsourcing in the UK National Health Service, and the extent of low-value care in German hospitals based on insurance claims data. These studies offer valuable evidence on individual policy instruments within specific national settings. However, most existing research remains focused on single-country cases or isolated policy tools. Comparative analyzes that integrate different governance models, institutional contexts, and recent policy developments across multiple health systems are still limited. In particular, few studies examine how combinations of policy instruments operate within diverse institutional settings and how they relate to system-level outcomes. To address this gap, this study conducts a systematic cross-national analysis of public hospital policy frameworks in five major economies and China. By integrating historical policy trajectories with recent reform developments, the study examines how different policy instruments function within varied institutional contexts and how they are associated with observable system-level outcomes.

**Figure 1:**
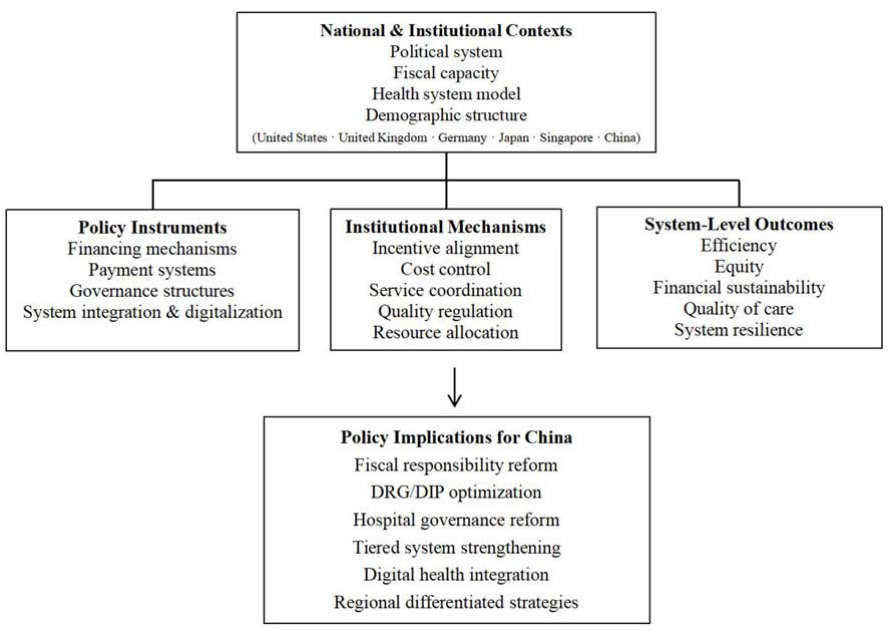
Analytical framework of the study on public hospital development policies across major economies and China.

Figure 1 provides an overview of the analytical framework of this study, illustrating how national contexts shape policy instruments, which in turn influence system-level outcomes and inform policy implications for China.

## 2 Policy trajectories and characteristics of public hospital support in major global economies

### 2.1 Policy framework for supporting public hospital development in the United States

#### 2.1.1 Institutional context

Table 1 shows the comparison of core features in public hospital support policies among major global economies and China. The United States has developed a mixed healthcare system centered on private insurance with public insurance as a supplement (1). Private health insurance serves as the core system, encompassing for-profit commercial insurance and non-profit private insurance. The public healthcare system includes multiple components such as Social Security Medicare, Medicaid, and the National Health Service model, each providing coverage for distinct populations (2). As a federal nation, healthcare policy in the United States is jointly managed by the federal and state governments, with local governments bearing significant responsibilities for implementation and funding allocation.

**Table 1:** Comparison of core features in public hospital support policies among major global economies and China.

**Country**	**Core funding mechanism**	**Primary governance model**	**Key policy features**	**Policy focus and challenges**
United States	Market-driven, with public insurance (Medicare/Medicaid) as the primary reimbursement channel	Indirect support, hospital autonomy, and high marketization	Services procured through public insurance programs rather than direct administrative funding and operational management.	Priorities: Expanding coverage, controlling costs, improving efficiency. Challenges: Fragmented coverage, inequities, and financial strain on some hospitals.
United Kingdom	Fiscally driven, with National Health Service (NHS) budget allocation	Government-led with internal marketization and performance governance	Tax-funded universal free healthcare; efficiency improvements through internal markets and performance-based payments.	Focus: Maintaining equity, controlling costs, improving service quality. Challenges: Significant fiscal pressure, long service waiting times, balancing fairness, and efficiency in reforms.
Germany	Social Health Insurance (mainstream) + Government Capital Investment (state governments)	Dual governance structure, corporate-style operation	Statutory health insurance covers operational costs, while state governments are responsible for capital investment, ensuring clear delineation of responsibilities.	Focus: Stabilizing the healthcare system, controlling cost growth, and promoting quality-based payments. Challenges: Coordinating federal and state responsibilities, addressing cost pressures from an aging population.
Japan	Social Health Insurance + Shared Central and Local Government Funding	Local governments take primary responsibility; institutional reform implemented	Introducing diagnosis procedure combination payment systems to control costs; local governments play a central role in public hospital investment and governance.	Focus: Addressing super-aging society, controlling healthcare costs, restructuring regional medical resources. Challenges: Central-local fiscal responsibility sharing; operational difficulties for hospitals in remote areas.
Singapore	Government subsidies + personal medical savings (Medisave system)	Corporate governance through group structures, with strong government oversight	Differentiated subsidies based on ward grades; formation of regional healthcare groups combining economies of scale with internal competition.	Focus: Balancing equity and efficiency, strengthening individual responsibility, enhancing system resilience. Challenges: Demands high precision in management, requires sustained containment of healthcare cost inflation.
China	Government-led + Basic Medical Insurance + Diversified Compensation	Separation of management and operation with a public welfare orientation	While upholding the government's responsibility for planning and investment, gradually introduce market mechanisms and performance management to explore a governance model suited to national conditions.	Focus: Eliminating drug-based compensation, establishing new compensation mechanisms, promoting high-quality development, and strengthening public welfare orientation. Challenges: Sustainability of fiscal investment, medical service pricing reform, implementation of tiered diagnosis and treatment, policy coordination.

The U.S. Affordable Care Act (ACA) provides the legal framework for establishing the federal healthcare programs Medicare and Medicaid. Medicare provides health insurance for individuals aged 65 and older and certain disabled groups, while Medicaid offers coverage for low-income populations. Together, they constitute the primary funding sources for public hospitals. However, significant variations in Medicaid implementation across U.S. states have led to disparities in payment standards and coverage scope, creating financial pressures for public hospitals in some states. Furthermore, as the U.S. population ages and healthcare demands increase, Medicare and Medicaid face sustainability challenges that may impact public hospitals' revenue and operational stability. Beyond relying on traditional programs like Medicare and Medicaid, the federal government has also advanced healthcare expansion and optimized payment methods through reforms such as the ACA, encouraging public hospitals to enhance service quality and operational efficiency. In recent years, public hospitals have also faced transformation pressures, shifting from traditional fee-for-service models to payment systems based on health outcomes. This requires hospitals to deliver more efficient, patient-centered care. Overall, U.S. public hospitals remain dependent on federal-state collaboration, seeking to ensure their critical role in serving vulnerable populations through the support of the healthcare system.

#### 2.1.2 Policy context

In 1965, the Medicare and Medicaid Act, enacted during President Johnson's administration, established the first federal public health insurance programs for the older adults (Medicare) and low-income individuals (Medicaid). This legislation provided public hospitals with a stable channel to serve these two major groups and receive corresponding compensation. During the 1970s, the U.S. economy faced the “stagflation” dilemma of sluggish technological innovation, slowing foreign trade growth, and economic stagnation, leading to persistently rising healthcare costs. Against this backdrop, the U.S. government sought to alleviate fiscal pressures through systemic adjustments. In 1982, the Reagan administration enacted the Deficit Reduction Act, followed by the nationwide implementation of a DRGs-based prepaid payment system in 1984. By establishing a standardized disease classification system, this reform enabled the Centers for Medicare and Medicaid Services (CMS) to precisely control reimbursement standards for healthcare services. Under this mechanism, healthcare providers' revenues became directly linked to treatment costs, compelling them to achieve budgetary balance through enhanced operational efficiency and optimized resource allocation.

The reform reduced Medicare inpatient costs by approximately 20% over 6 years (3), while significantly improving overall hospital service efficiency. The U.S. government has long sought to balance expanding healthcare accessibility with controlling medical costs. However, the 2008 global financial crisis plunged the U.S. economy into crisis once more. Widening income inequality, rising unemployment, and declining incomes triggered a series of social issues. The shortage of market-based public goods became more severe, leading to inadequate health insurance coverage. By 2010, nearly 50 million Americans remained uninsured. Facing social pressures stemming from healthcare accessibility, the Obama administration advanced healthcare reform by strengthening the government's role, aiming to alleviate social hardships and consolidate the foundation for social stability (4). The ACA, formally signed into law in March 2010, marked a new phase in U.S. healthcare reform. In 2025, the CMS continued to adjust Medicare hospital payment policy through annual rulemaking, with updates finalized in the Calendar Year 2026 Hospital Outpatient Prospective Payment System (OPPS) and Ambulatory Surgical Center (ASC) Payment System final rule fact sheet (5). CMS also strengthened hospital price transparency requirements, requiring hospitals to post actual, consumer-usable prices in standardized formats (6).

#### 2.1.3 Characteristics

The U.S. support policies for public hospitals exhibit distinct characteristics: market mechanisms serve as the primary driver, supplemented by public programs, with adjustments made dynamically in response to socioeconomic conditions. The core of these policies lies not in direct administrative funding or operational management of public institutions, but rather in indirectly providing financial resources and guiding hospital behavior through the establishment and refinement of public healthcare security systems. The federal government provides crucial financial backing to public hospitals through programs like Medicare and Medicaid, ensuring they can deliver essential healthcare to low-income and older adults populations. However, these subsidies offer only limited support. While reliant on public funds, public hospitals must also confront market-driven operational pressures and competitive environments. Against this backdrop, many public hospitals have progressively adopted market-oriented management models, implementing strategies similar to those of private hospitals. This market-oriented transformation helps public hospitals improve operational efficiency, reduce financial dependence, and enhance competitiveness. Overall, U.S. support policies for public hospitals essentially constitute an indirect support model deeply integrating market incentives and government regulation, underpinned by public finances and utilizing insurance payments as tools. This approach ensures the provision of basic healthcare services while enhancing public hospitals' adaptability and sustainability within the healthcare market.

### 2.2 The policy framework supporting public hospital development in the United Kingdom

#### 2.2.1 Institutional context

The formation and development of the UK's public hospital system has been profoundly shaped by the National Health Service (NHS), with its core foundation rooted in a tax-funded universal healthcare model. Since the implementation of the NHS in 1948, the UK has established a public healthcare system based on full state funding. The government centrally finances and allocates the overall budget, providing universal free healthcare services. The UK government funds public hospitals through general taxation, ensuring citizens incur no direct medical costs when seeking care and achieving the system's objectives of universal coverage and provision based on need.

Within the NHS framework, public hospitals form the core of the healthcare delivery system, with their operational funding primarily derived from annual government budget allocations. Regarding payment mechanisms, the UK has transitioned from a purely annual budget model toward more efficiency-focused approaches. Examples include implementing a pay-for-performance system with centrally set uniform prices, and actively exploring integrated care and value-based payments to incentivize hospitals to deliver more efficient, patient-centered services. Thus, the UK's public hospital system centers on the NHS. Supported by full state funding, it has established a public healthcare system grounded in principles of fairness and universal access, ensuring basic medical coverage for all citizens. Simultaneously, it undergoes continuous reform to address fiscal pressures and structural shifts in health demands.

#### 2.2.2 Policy context

The institutional foundation of Britain's modern public hospital system traces back to the 1948 National Health Service Act. This legislation established the basic framework for the NHS, creating a tax-funded, government-managed universal healthcare model. This institutional arrangement made the UK one of the earliest nations to achieve universal free healthcare. Primary care services are delivered by community general practitioners (GPs), while secondary care is provided by regional general hospitals and central specialist hospitals. The patient pathway typically begins with an initial consultation at a GP practice, requiring a referral letter to access specialist care within the hospital system. In the 1990s, the UK government initiated internal market reforms. Under the 1990 National Health Service and Community Care Act, public hospitals were restructured into NHS Trusts with independent legal status, overseen directly by the Department of Health. Under this framework, hospital directors were publicly recruited by boards of trustees. Serving as the highest decision-making bodies, these boards were responsible for strategic planning and management oversight, establishing a governance structure combining government macro-guidance with hospital autonomy. Policies during this phase promoted modernized hospital management, market-oriented operations, and performance-based service delivery, laying the foundation for subsequent performance assessment systems (7).

Entering the early 21st century, the UK government released the landmark NHS Plan and NHS Modernization Strategy, initiating a phase of healthcare service modernization and information technology development. In 2003, the UK government passed the Health and Social Care Act, giving rise to the new Foundation Trust model. These hospitals enjoy greater financial and managerial autonomy and implement social oversight and democratic participation through a governance mechanism involving councils with representatives elected by residents. This move signified the transformation of UK public hospital governance structures from direct state control to delegated autonomy and shared social governance (8). Concurrently, starting in 2004, the NHS implemented the Quality and Outcomes Framework (QOF) for performance evaluation. By directly linking assessment outcomes to financial subsidies, QOF continuously drove improvements in healthcare service quality. Evidence on patient-reported outcomes remains limited, but recent analysis suggests that higher QOF achievement is associated with a small yet statistically significant improvement in health-related quality of life (measured using EQ-5D-5L) among adults with long-term conditions in England. These findings indicate that pay-for-performance incentives may translate into modest gains in patient-perceived health status, although the magnitude appears limited (9).

Since 2010, under dual pressures of fiscal austerity and rising healthcare demand, the UK government has intensified cost control and performance optimization. Concurrently, the 2012 Health and Social Care Act signaled deepened market-oriented reforms by substantially encouraging private healthcare providers to bid for NHS service contracts. UK public hospitals began appropriate collaborations with private entities to alleviate financial strain and enhance system resilience. The policy focus shifted from mere expansion to enhancing quality and efficiency, optimizing governance, and controlling costs, driving public hospitals toward institutionalized, refined, and sustainable development while maintaining their public attributes (10).

The COVID-19 pandemic exposed and exacerbated inherent vulnerabilities within the NHS system, such as an aging population and increasing burdens of chronic diseases. Consequently, the UK government launched the NHS Ten-Year Plan to restructure the healthcare system, focusing on technological upgrades and preventive medicine. The landmark Health and Care Act 2022 promotes the establishment of an integrated care system. This aims to break down barriers between institutions like the NHS, local governments, and social services, replacing past internal competition with collaborative partnerships to more effectively address long-term challenges such as population aging and chronic disease management.

In 2025, NHS England further operationalized elective care reform through a national delivery program that emphasizes pathway redesign, expanded diagnostic access, and productivity-oriented incentives for provider organizations. The framework prioritizes straight-to-test pathways, outpatient reform to reduce low-value follow-ups, and stronger performance oversight across trusts, linking implementation to funding design and system management expectations (11). Concurrently, the Government's 2025 mandate to NHS England reaffirmed a strategic focus on reducing elective waiting times and improving patient experience, highlighting the need to deploy resources more efficiently while maintaining quality and safety. Together, these measures indicate an incremental reform approach centered on operational performance, demand management, and delivery-system accountability rather than large-scale structural reorganization (12).

Consequently, the evolution of supportive policies for UK public hospitals reflects a shift in core policy tools. While maintaining consistent government financial investment and institutional frameworks, the approach has progressively transitioned from direct government management to the introduction of internal market mechanisms, ultimately developing into a hybrid model emphasizing performance evaluation and autonomous governance. Its defining characteristic lies in the government's sustained commitment to public financial responsibility, coupled with ongoing adjustments to governance and payment mechanisms. Through institutional innovation, this approach enhances the healthcare system's efficiency and equity, building a more resilient, effective, and future-ready healthcare infrastructure.

#### 2.2.3 Characteristics

The UK's public hospital system operates under the unified framework of the NHS, primarily tax-funded, establishing a healthcare service model characterized by deep government intervention and clear public financial accountability. The government assumes integrated roles in planning, funding, and oversight, directly allocating resources through the central budget to ensure universal access and national uniformity of healthcare services. This system avoids rigid bureaucracy. Post-1990s reforms introduced mechanisms to enhance resource allocation efficiency, including separation of purchaser and provider, establishment of foundation trusts, and implementation of outcome-based payments and quality performance management. Policy evolution has upheld tax-funded universal free healthcare while adjusting governance and incentive structures to prevent inefficiency, forming a unique model combining planned coordination with market-based allocation.

The core insight from the UK experience lies in demonstrating the long-term dynamic process through which public service systems achieve both equity and efficiency. Even under government leadership, a public hospital development model that balances equity, efficiency, and quality improvement can be constructed through sound governance structures and performance management tools.

### 2.3 Germany's policy framework for supporting public hospital development

#### 2.3.1 Institutional context

German hospital revenues primarily derive from statutory health insurance payments, while capital investments rely on government funding, particularly state budgets, forming a unique healthcare system anchored by social health insurance and coordinated through corporate governance and federalism. This system features clear delineation of responsibilities. Statutory health insurance organizations are primarily responsible for funding, covering an overwhelming majority of insured individuals. Federal legislative bodies take charge of establishing the overarching legal framework and setting quality standards for the healthcare system. Meanwhile, state governments are tasked with bearing the capital expenditures for public hospitals, which include infrastructure construction and the procurement of major medical equipment. Such an arrangement gives rise to a dual governance structure that effectively separates daily operations from capital investment.

Germany's healthcare system operates under a mixed-ownership model with a diverse range of service providers. There are four types of institutions within this system, including private clinics and general hospitals. Based on their sponsoring entities and operational nature, hospitals can be classified into three categories. Firstly, public hospitals are directly invested in and managed by the government (or are affiliated with universities). Secondly, non-profit private hospitals are operated by entities such as churches. Thirdly, for-profit private hospitals are managed by private capital, although they may also receive government investment. Public and non-profit private hospitals play a dominant role in the sector, delivering the bulk of primary healthcare services.

The healthcare security system in Germany is centered around statutory health insurance, which is supplemented by private health insurance. Statutory health insurance is legislated at the national level and administered by non-profit health funds. Insured individuals have the freedom to choose their preferred health fund provider. Private insurance addresses specialized needs beyond statutory coverage. In the first half of 2020, statutory insurance covered 88.2% of Germany's population, maintaining high penetration rates (13). Fiscal compensation employs a dual-track system based on DRGs. Government budgets cover hospitals' long-term investment costs, while statutory insurance pays service fees according to DRG standards. In summary, Germany's healthcare service model primarily relies on public and non-profit hospitals to deliver basic medical services. Through government macro-governance, hospitals operate autonomously while leveraging market incentives for efficient management. This approach lays a solid foundation for the sustainable development of public hospitals and the efficient operation of the overall healthcare system (14).

#### 2.3.2 Policy evolution

The development of supportive policies for German public hospitals exhibits distinct phased characteristics. The enactment of the Workers' Sickness Insurance Act in 1883 marked the beginning of over a century of continuous expansion in Germany's health insurance coverage. In the 1970s, the Hospital Financing Act established a “dual-tier system” funding framework, clarifying hospital funding sources: state governments handle capital investments while health insurance funds cover operational costs. This accelerated public hospital development, but later faced challenges due to insufficient fiscal investment, leading to public hospitals' reliance on government funding, mounting debt, and widening deficits. Although there is no nationally harmonized time series reporting the exact debt ratios of public hospitals under Germany's dual financing regime, empirical studies and industry analyzes indicate that the model has long been associated with persistent investment shortfalls at the state level, leading many hospitals to cover capital expenditure from operating revenues and external financing sources rather than full state subsidies (15). These financing pressures are understood to contribute to increasing financial burdens for public hospitals, particularly in capital-intensive investments.

At the dawn of the 2000s, Germany's healthcare costs soared swiftly, mainly propelled by structural changes on both the demand and supply fronts. On the demand side, universal coverage under statutory health insurance enhanced public access to medical services, stimulating demand for high-quality, comprehensive care. Concurrently, population aging shifted disease patterns toward chronic conditions, with older adults patients' long-term care and integrated treatment needs placing sustained pressure on healthcare expenditures. On the supply side, advances in medical technology and their application improved diagnostic and treatment standards, but their high costs drove up overall healthcare expenditures. Facing rising costs due to chronic diseases, the German government enacted the Digital Healthcare Act in 2019, emphasizing the role of mobile health apps in chronic disease management, particularly diabetes. Additionally, Germany introduced the G-DRG payment system to control cost growth. This system enables precise case grouping through comprehensive analysis of multidimensional data, including disease diagnoses, supporting health insurance payments and hospital management. It marks a shift in hospital compensation models from fee-for-service to a prepaid system.

However, traditional DRG systems incentivized hospitals to prioritize volume over quality. In 2024, the German Bundestag adopted the Hospital Care Improvement Act, which came into force in January 2025. The reform marks a shift away from the traditional DRG-based reimbursement model toward a hospital classification system based on service capacity. Under the new framework, hospitals are grouped according to their clinical capabilities, staffing qualifications, and compliance with defined quality standards. Public funding is increasingly linked to structural capacity and quality requirements rather than service volume alone. The reform aims to limit unnecessary hospital expansion, strengthen regional care coordination, and improve the quality of complex and emergency services. In brief, Germany's public hospital support strategy broadens social health insurance, balances cost and quality, and uses digital healthcare to optimize resources, enhance services, and improve access, showing adaptability and coordination.

#### 2.3.3 Characteristics

The German government's support policies for public hospitals exhibit distinct characteristics. In terms of institutional safeguards, the government clearly delineates the responsibilities of statutory health insurance and public finances: the insurance system covers hospitals' daily operational costs, while governments at all levels handle capital investments. This design ensures the financial stability of public hospital operations, laying a solid foundation for their sustainable development. Regarding the allocation of authority and responsibility, Germany leverages the advantages of its federal system to establish a scientifically structured division of labor. The federal government provides top-level design, legislating clear quality standards and service norms; state governments implement capital investments and allocate resources based on regional needs. This arrangement ensures the uniformity of the national healthcare service system while accommodating local variations, demonstrating the wisdom of tiered governance.

In terms of policy evolution, Germany possesses the capacity for continuous optimization and dynamic adjustment. From establishing a dual governance mechanism to introducing the DRG system for cost control, and then driving the shift from fee-for-service to pay-for-performance, the German government has consistently adjusted its support policies in response to developmental stages and practical needs, demonstrating the resilience of institutional self-improvement. In summary, the German government's support for public hospitals goes beyond mere financial investment; it encompasses a comprehensive support system covering institutional frameworks, responsibilities, operations, and policy optimization, offering significant reference value for deepening healthcare reform in China.

### 2.4 Japan's policy framework for supporting public hospital development

#### 2.4.1 Institutional context

Japan operates under a social insurance-based healthcare model with substantial government involvement. The Ministry of Health, Labor and Welfare (MHLW) formulates national health security policies and guides the implementation of healthcare plans across Japan's 47 prefectures. Hospitals are broadly classified into three categories according to ownership, namely government-operated public hospitals, quasi-public hospitals, and privately-run hospitals (16). Public hospitals, predominantly operated by local governments, handle primary care and emergency services, playing a crucial role in grassroots healthcare and disease prevention. Private hospitals concentrate on high-end medical care, specialized treatments, and innovative technologies, demonstrating strong market competitiveness. Despite competition, both sectors are regulated by policies and laws, complementing each other to promote efficient allocation of medical resources and enhance service quality. Japan implements a universal health insurance system. Since 1961, the government has provided comprehensive medical coverage for all citizens through mandatory health insurance plans, ensuring equitable and universal access to healthcare resources.

Japan's social health insurance system primarily consists of two components: Employee Health Insurance and National Health Insurance. Employee Health Insurance covers industrial workers, civil servants, and their dependents, while National Health Insurance primarily serves agricultural workers, freelancers, and retirees. Funding follows the principle of shared responsibility, with costs borne jointly by national finances, employers, and individuals (17). The universal healthcare system covers ordinary residents, the older adults, low-income groups, and other vulnerable populations, providing essential medical services that effectively reduce the public's healthcare costs and promote universal access to medical care. Overall, within the institutional framework of universal healthcare alongside coexisting public and private hospitals, Japan's public hospital system delivers universal and equitable medical coverage to its citizens, ensuring efficient and sustainable healthcare services.

#### 2.4.2 Policy context

After the conclusion of the major global conflict in the mid-20th century, Japan faced the dual challenges of reconstruction and scarce medical resources. Insufficient healthcare resources and disparities in medical services between urban and rural areas became pressing issues requiring urgent solutions. To address this, the Japanese government established universal access to healthcare as a long-term development goal and promoted the construction of public hospitals. By building extensive medical infrastructure, it laid the foundation for implementing the universal healthcare system.

In 1956, Japan's Ministry of Health and Welfare formulated the Four-Year Plan for Nationwide Universal National Health Insurance, proposing the goal of establishing a welfare state centered on universal health insurance and universal pension schemes. Subsequently, the government made significant revisions to the Health Insurance Act and the National Health Insurance Act, establishing a system where patients bear a portion of medical costs and mandating enrollment in National Health Insurance. The National Pension Act was enacted in 1959, establishing the welfare pension system. By 1961, Japan's universal healthcare system was largely in place. As the population aged, pressure on medical insurance funds grew. To address this challenge, the Japanese government built upon the existing employee health insurance and resident health insurance systems to create a health insurance system specifically for the older adults, complemented by a long-term care insurance system.

In the 1980s, the coverage of the older adults health insurance expanded, but rising medical costs continued to strain the insurance fund. To address this, Japan enacted the Health and Medical Services for the Older Adults Act in 1982, creating the Older Adults Health Insurance system. This system alleviated financial pressure through cross-fund risk sharing and patient co-payment mechanisms for older adults patients (18). Concurrently, a long-term care insurance system was established, separating long-term care costs from routine medical expenditures for independent management. From the 1980s to the 1990s, confronting dual challenges of fund pressures and rising medical costs, the Japanese government shifted toward policies emphasizing cost control, drug price management, and expenditure restraint. This optimized resource allocation while enhancing healthcare efficiency and quality (19). Concurrently, to address long-term care needs for the older adults, Japan established the Long-Term Care Insurance System in the early 2000s, alleviating pressure on the health insurance fund (20). To curb overmedicalization, Japan formally introduced the Diagnosis Procedure Combination/Payment by Diagnosis Group (DPC/PDPS) system in 2003. By setting daily standard points for specific conditions, this system reduces financial incentives for prolonging hospital stays, effectively curbing overmedicalization and improving healthcare efficiency (21). Evaluations of Japan's DPC/PDPS indicate a sustained reduction in hospital length of stay following its implementation, reflecting improved inpatient efficiency under prospective payment arrangements.

Official assessments by the MHLW report that DPC hospitals consistently exhibit shorter average lengths of stay than fee-for-service hospitals, with a continuing downward trend over time (22, 23). Empirical studies further provide disease-specific evidence of this effect. For acute myocardial infarction, the introduction of DPC/PDPS has been associated with an average reduction in length of stay of approximately 2.3 days, without a corresponding increase in short-term mortality (24). In patients with hip fractures, implementing hospitals reduced average length of stay from 30.1 days to 23.5 days, whereas non-implementing hospitals showed only a marginal decline from 38.5 to 36.4 days over the same period (24). In addition, longitudinal analyzes of surgical cohorts, including patients undergoing cancer-related procedures, have documented a gradual annual decrease of around 0.5 days in length of stay after the adoption of DPC/PDPS, with no significant rise in readmission rates (25). Together, these findings suggest that the DPC/PDPS system has contributed to measurable reductions in inpatient stay across multiple clinical conditions, supporting its role in enhancing hospital efficiency in Japan.

Entering the 21st century, addressing the widespread operational challenges faced by public hospitals, the Japanese government promoted the transformation of public hospitals toward a corporate governance structure. Measures included consolidating redundant institutions, introducing market-oriented operational models, and forming regional medical groups to enhance the management efficiency and market adaptability of public hospitals. Concurrently, internal governance structures were strengthened by implementing a president-responsibility system under board leadership, alongside the introduction of corporate accounting and performance evaluation tools to improve financial transparency and management standards. After 2015, Japan's policy shifted toward restructuring healthcare supply on a regional basis. This emphasized adjusting bed functionality planning according to population trends and disease patterns, promoting networked collaboration among hospitals to enhance system resilience and efficiency. Simultaneously, public hospitals were required to play a central role in medically underserved areas, emergency response systems, and public health crises, ensuring essential medical services during emergencies.

The COVID-19 pandemic further tested the effectiveness of these policies, prompting the government to make corresponding adjustments in short-term financial support, the designated hospital system, and public health preparedness. Japan is advancing regional healthcare restructuring under the Regional Medical Care Vision framework toward the 2025 planning horizon. Prefectural governments are required to align service capacity and hospital bed functions with projected demand (26). The framework reinforces functional differentiation between acute, recovery, and long-term care. Bed function differentiation has become a key implementation mechanism to optimize resource allocation in the context of population aging and to reduce inefficiencies associated with mismatched bed utilization (27). Overall, the reform emphasizes planning-based capacity management and service coordination rather than relying solely on payment incentives. In summary, despite post-war public hospitals' poor performance, Japan quickly adjusted its healthcare model. Through policies like universal health insurance, payment reforms, and hospital restructuring, it has sustained basic healthcare services and medical quality.

#### 2.4.3 Characteristics

A defining feature of Japan's public hospital development lies in the meticulous design of its payment system and the strengthened responsibilities of local governments. The refinement of the payment system is primarily reflected in the deep implementation of the DRG payment system and the DPC/PDPS. Through this series of sophisticated payment mechanisms, Japan has been able to allocate medical resources in a more scientific and precise manner, effectively enhancing the quality and efficiency of healthcare services. Simultaneously, local governments play a pivotal role in the development of Japan's public hospitals. Local governments not only shoulder substantial capital investment responsibilities for public hospitals, covering critical areas such as hospital infrastructure construction and medical equipment procurement, but also actively engage in optimizing hospital governance structures and enhancing operational efficiency. Through initiatives like promoting the incorporation of public hospitals, introducing corporate accounting management systems, and implementing performance evaluation mechanisms, local governments have further strengthened their management responsibilities, laying a solid foundation for ensuring the service quality and economic sustainability of public hospitals. This clear division of responsibilities enables local governments to play an irreplaceable role in ensuring the stable operation and fairness of public hospitals. Facing severe challenges such as market competition and population aging, public hospitals can achieve better responses and long-term sustainable development with the firm support of local governments.

### 2.5 Singapore's policy framework supporting public hospital development

#### 2.5.1 Institutional context

Singapore operates a dual-track healthcare system encompassing both government-run public and private medical institutions, with public hospitals playing a dominant role. The government funds this system through taxation, directly subsidizing the cost of services provided by public institutions. Citizens pay subsidized rates for medical care, with subsidy levels closely tied to their ability to pay, determined through means testing and adjusted based on ward classification. For instance, in Singapore's public hospitals, inpatient ward subsidies are allocated through a means-tested framework administered by the Ministry of Health. Government subsidies apply to subsidized ward classes such as Class C and B2, with eligible Singapore Citizens receiving up to 80% of ward charges depending on per capita household income. By contrast, higher-amenity wards such as Class A (single bed private rooms) do not attract government subsidies, reflecting the policy's progressive distribution of public support according to service level and patient financial capacity (28). This design ensures broad accessibility to essential healthcare services while preventing high-income individuals from disproportionately occupying public medical resources.

Singapore's social security system centers on the Central Provident Fund (CPF) scheme, which mandates employers and employees to contribute a percentage of wages into individual accounts through legislation. This compulsory savings program addresses citizens' long-term expenditure needs for retirement, healthcare, education, and more. Based on this, Singapore has established a 3M healthcare system: First, the MediSave scheme is a mandatory personal medical savings program requiring employed individuals to allocate a portion of their income to the MediSave sub-account within their CPF account. This fund covers hospitalization costs, certain expensive outpatient treatments, and long-term care expenses for the account holder and their immediate family members (29). It emphasizes personal responsibility for health while providing stable funding for the healthcare system; Second, the MediShield Life scheme provides mandatory universal basic coverage against large hospital bills and selected high-cost treatments (30). It mitigates individual financial risks through social mutual aid, with premiums payable from MediSave accounts to ensure both accessibility and compulsory participation. Third, the MediFund serves as the ultimate safety net. Established by the government as a donation fund, its returns subsidize impoverished citizens who remain unable to cover medical expenses after utilizing MediSave and MediShield Life, ensuring no citizen is denied essential healthcare due to financial constraints.

Following the corporatization of Singapore's public hospitals, government regulation of healthcare service pricing triggered competition among hospitals for patient resources, leading to imbalanced healthcare resource allocation and hindering the development of small and medium-sized hospitals. To counteract the negative effects of excessive competition, the government promoted group-based reforms within the public healthcare system. This consolidated national medical institutions into three major regional healthcare groups: the Singapore Health Services (SHS), the National University Health Services (NUHS), and the National Healthcare Group (NHG). This restructuring facilitated resource integration and collaboration, providing more coordinated healthcare services to community residents.

In summary, Singapore's public hospitals operate within a hybrid public-private system characterized by government subsidies and the 3M framework, where responsibilities are shared. Government subsidies ensure equity and accessibility, compulsory savings reinforce personal responsibility and cost awareness, social medical insurance mitigates major illness risks, and medical assistance ultimately fortifies the social safety net. This sophisticated institutional design establishes clear financial rules and incentive mechanisms for efficient hospital operations, forming the foundation for effective policy implementation.

#### 2.5.2 Policy framework

Singapore's public hospital system adopts a model integrating government guidance with market mechanisms. It employs indirect means such as legislative regulation and institutional design to steer hospital development at the macro level, rather than direct administrative intervention in operations (31). This governance philosophy became prominent during the 1980s healthcare reforms, which began by restructuring the financing system. In 1984, Singapore established the Medisave scheme, subsequently refining critical illness insurance (Medishield Life) and medical assistance (Medibond) mechanisms. This formed a healthcare financing framework with clear responsibilities and multi-tiered protections, rationally distributing obligations among stakeholders and laying the groundwork for deepening public hospital reforms. To address public hospitals' inefficiency and sluggish market responsiveness, the Singaporean government implemented corporatization reforms. The restructured public hospitals became autonomous entities, while government subsidies shifted to a demand-oriented approach. This clarified target groups and coverage scope, enhancing the precision and efficiency of subsidies.

Following the completion of hospital organizational restructuring in the 1990s, the Singaporean government focused on refining the 3M healthcare security system, establishing an initial framework for tiered healthcare system and treatment alongside stratified subsidies. Within the refined payment system reforms, subsidies were strictly linked to ward grades, forming a differentiated subsidy matrix ranging from Grade A (0%) to Grade C (80%). This guided patients to select wards based on their payment capacity, achieving cross-subsidization among different income groups. Simultaneously, targeted subsidies for specific demographics were implemented through means testing. Patients bypassing referrals to directly access tertiary hospitals faced lower subsidies and higher out-of-pocket costs, establishing an economic incentive to steer patients toward primary care in communities. This laid the institutional foundation for the tiered healthcare system. Singapore established six Regional Healthcare Systems (RHS) centered on health, forming a horizontally integrated and vertically interconnected healthcare service system to alleviate overmedicalization and hospitalization issues (32).

Since 2020, Singapore's public hospital policy focus has shifted toward system resilience, digital transformation, and comprehensive health management, with further deepening of group-based and tiered healthcare systems. Digital transformation has become central, with upgraded data-sharing platforms within and between healthcare groups providing technological support for integrated care and telemedicine, making tiered healthcare system and two-way referrals more efficient. Under the RHS 2025 strategy, Singapore has accelerated digital integration across regional healthcare systems. The National Electronic Health Record (NEHR) platform now supports real-time data sharing among public hospitals, primary care clinics, and long-term care institutions (33). By 2025, all nine private hospitals in Singapore have committed to sharing patient health records on the national NEHR platform to facilitate continuity of care across providers (34). This digital upgrading enhances referral efficiency, chronic disease management, and population health monitoring, particularly for aging communities.

#### 2.5.3 Characteristics

The Singapore model exemplifies a sophisticated blend of state-led initiatives and market mechanisms, demonstrating exceptional precision in governance through subsidy design and group management. By pursuing three core policy pillars, namely public hospital group and corporate reforms, tiered healthcare system development, and a refined dynamic subsidy matrix, it systematically addresses public hospitals' efficiency, autonomy, resource allocation, and equitable accessibility. For China, currently in a critical phase of deepening healthcare reform, this model offers invaluable reference points and insights on balancing government-market relations, designing precise fiscal subsidies and payment mechanisms, and advancing comprehensive public hospital reforms alongside tiered healthcare implementation.

## 3 Policy framework and measures supporting the development of public hospitals in China

### 3.1 Policy framework

China's public hospital development path exhibits distinct phased characteristics, with the state introducing a series of supportive policies to ensure reform advancement. In the early years after the founding of the People's Republic, China faced severe shortages of healthcare resources, with only 3,670 healthcare institutions and 505,000 technical personnel, grappling with challenges such as high incidence of infectious diseases and low life expectancy. The core objective during this phase was establishing a basic healthcare service delivery system to ensure service accessibility. Through a government-led planning model, the state built a welfare-oriented healthcare system integrating prevention and treatment, while vigorously cultivating healthcare professionals. Public hospitals dominated the primary healthcare service system, making significant contributions to meeting basic medical needs, controlling infectious disease outbreaks, reducing infant and maternal mortality rates, and increasing life expectancy. However, constrained by economic development conditions, government fiscal investment was limited, and healthcare service pricing remained low, leaving most public hospitals operating at a deficit. In 1954, the Ministry of Health approved the implementation of a drug markup policy, which later evolved into the “subsidizing hospitals through drug sales” system. This profoundly influenced the operational model and development trajectory of public hospitals.

Following reform and opening-up, the disease spectrum shifted from infectious to chronic illnesses, rendering the planned-economy healthcare system inadequate for diverse health needs. In 1985, the State Council endorsed the Ministry of Health's Report on Several Policy Issues Concerning Healthcare Reform, initiating healthcare reforms with measures including increased health funding and policy liberalization. In 1989, China issued the Opinions on Issues Concerning the Expansion of Medical and Health Services, introducing market incentive mechanisms such as the contract responsibility system and limited opening of fee-based services. Concurrently, the Measures for the Graded Management of Medical Institutions (Trial) was promulgated, aiming to optimize the healthcare system structure and rationally allocate resources through a tiered evaluation system. During this phase, the efficiency of healthcare resource supply and technical standards improved. However, a decline in government investment and intensified competition shifted hospital service models from “combining prevention and treatment” to “treatment-centered care,” weakening the public welfare orientation.

To address these new challenges, China introduced multiple policies. The 1997 Decision of the Central Committee of the Communist Party of China and the State Council on Health Reform and Development explicitly required healthcare institutions to prioritize social benefits. In 2000, the General Office of the State Council forwarded opinions proposing to transform the operational mechanisms of public medical institutions and expand their autonomy. Subsequent supporting policies encouraged the marketization of medical services, but also led to a dilution of the public welfare nature of public medical institutions. Entering the 21st century, the policy direction became clear. A 2005 Ministry of Health document reaffirmed the public welfare nature as the fundamental attribute of public medical institutions. The 2008 Hospital Management Evaluation Guidelines established a performance evaluation system to drive the transition toward public welfare-oriented management. The new round of healthcare reform launched in 2009 systematically advanced pilot reforms in public hospitals from 2009 to 2011. These reforms eliminated markups on drugs and medical consumables, established long-term operational mechanisms, and built a new hospital management system. The government clarified its responsibilities by covering investments in public hospital infrastructure and offering special subsidies for public health tasks. It strictly controlled the scale of public hospital construction, implemented the separation of medicine and treatment, addressed revenue reductions or losses through multiple channels, and encouraged the exploration of medical service pricing negotiation mechanisms.

From 2012 to 2015, the government implemented its responsibility for hospital administration, advanced compensation mechanism reforms, controlled medical cost growth, promoted the separation of government functions from hospital operations, and accelerated the reform process. From 2016 to 2020, a modern hospital management system was gradually established, medical service prices were adjusted, a dynamic adjustment mechanism was created, pricing for certain medical services was liberalized, and staffing, personnel, compensation systems, and hospital evaluation mechanisms tailored to the industry's characteristics were established. From 2021 to the present, public hospitals have entered a phase of high-quality development. Initiatives ranging from the State Council General Office's Opinions on Promoting High-Quality Development of Public Hospitals to the Evaluation Indicators for High-Quality Development of Public Hospitals (Trial) have vigorously advanced the integration of clinical operations and management, enhancing operational efficiency. The system of hospital directors accountable to Party committees has been fully implemented, performance evaluations deepened, and comprehensive reform demonstrations and modern hospital management pilot programs advanced.

At the subnational level, Jiangsu and Zhejiang provinces have established public hospital groups to promote resource sharing, electronic medical record interoperability, and the development of regional medical centers. These reforms have strengthened referral coordination and reduced the duplication of high-cost services, improving the efficiency of service delivery within local health systems. The Sanming healthcare reform model has been widely promoted nationwide. Key features include eliminating drug mark-ups, introducing bundled payment mechanisms, and reforming physician salary systems to emphasize professional value over service volume. Empirical evidence shows that Sanming achieved sustained control of medical expenditure growth while improving service efficiency. The reform model has been replicated nationwide, deepening reforms in medical service pricing and personnel compensation systems, refining the tiered healthcare system, and promoting equitable distribution of high-quality medical resources. The focus has shifted to payment systems and service models, with pilot reforms in medical insurance payment methods driving the intrinsic development of medical services. Public hospital reform has thus entered a new era of comprehensive governance.

### 3.2 Supportive measures

The government ensures the smooth transition of this complex system through a series of coordinated and complementary policy supports. Regarding the clarification of reform direction, since the launch of the new healthcare reform in 2009, a series of top-level design documents have explicitly stated that upholding public welfare is the fundamental principle of public hospital reform (35). This guides hospitals to shift from a disease-centered approach to a people's health-centered approach, establishes high-quality development as the core task for public hospitals, and promotes a transition from scale expansion to quality-driven development through high-level conferences and policy guidance, focusing on quality, efficiency, and innovation. At the compensation mechanism level, the government concurrently established a multi-channel compensation system, including adjusting medical service pricing, increasing government subsidies, and reforming medical insurance payment methods. At the system integration level, the advancement of the tiered healthcare system alongside the substantive operation of medical consortiums and medical federations further optimized the spatial distribution and utilization efficiency of medical resources, reshaping an orderly healthcare system (36).

### 3.3 Existing challenges

The reform process has also highlighted certain systemic challenges. First, regarding compensation mechanisms, while the comprehensive abolition of the policy of subsidizing hospitals through drug sales has effectively reduced drug costs, it has introduced new issues such as rising medical service fees. This has also placed operational pressure on some hospitals facing policy-induced losses. Second, regarding price formation mechanisms, the long-standing issue of severe divergence between technical service pricing and actual costs remains unresolved. This has dampened healthcare professionals' sense of professional value and constrained the endogenous motivation for medical technology innovation. Third, regarding system integration efficiency, problems persist in the practical implementation of tiered healthcare system. The core reasons likely lie in inadequate primary healthcare service capabilities and the lack of effective benefit-sharing and risk-sharing mechanisms between medical institutions at different levels. This has prevented timely reversal of the siphoning phenomenon where large hospitals attract excessive resources and the resulting irrational resource allocation patterns (37). Finally, at the governance system level, coordination among different policy actors faces certain blockages, resulting in fragmented policy implementation. Combined with China's regional disparities, this leads to significant heterogeneity in the implementation methods and outcomes of reform policies, posing severe challenges to building a unified, balanced, and high-quality development system.

## 4 Similarities and differences between public hospital support policies in major global economies and China

### 4.1 Common features

In the healthcare systems of major global economies, the survival and development of public hospitals hinge on supportive government policies. Despite variations in political systems, economic levels, and cultural backgrounds, comparative analysis uncovers significant commonalities. Governments universally acknowledge their fundamental responsibility to provide basic healthcare guarantees, especially within the public hospital system. Whether in the United Kingdom with its National Health Service model, Germany with its social health insurance system, or the United States with its highly developed market mechanisms, governments ensure that public hospitals play an irreplaceable role in addressing public health crises, serving vulnerable populations, and delivering universal healthcare services. This approach safeguards the overall stability and equitable service delivery of the healthcare system.

### 4.2 Differences

#### 4.2.1 The United States leans toward marketization, while the United Kingdom leans toward fiscal dominance

From a fiscal investment perspective, the financial support mechanisms for public hospitals vary significantly across countries. In the United States, financial support for public hospitals is targeted and limited. Federal direct funding is minimal, with compensation primarily provided indirectly through healthcare security systems. For instance, the Medicaid Act subsidizes hospitals treating low-income or uninsured patients, safeguarding their safety net function, with funds precisely allocated to address shortcomings. The UK's public hospitals operate under a centralized, fiscally dominant system. Within the NHS, funding primarily originates from central general taxation. The central government allocates budgets at the macro level, with funds disbursed down through tiers to local authorities. Local authorities then distribute resources based on hospitals' actual needs and capabilities, ensuring funding stability, optimizing resource allocation, and promoting equity.

China's public hospital financing has undergone significant transformations. From the early years of the People's Republic through the planned economy era, public hospitals relied entirely on government subsidies. Following the reform and opening-up, fiscal support diminished, leading hospitals to operate on a self-financing basis and exacerbating the “drug-subsidized medicine” issue. After the 2009 healthcare reform, the government increased investments to support infrastructure and public health services. The fiscal support models of the United States, the United Kingdom, and China exhibit distinct differences. The U.S. emphasizes precise targeting and responsibility sharing, the UK stresses central government leadership and unified planning to ensure fairness, while China prioritizes universal coverage and government leadership. These approaches directly shape the divergent development models of public hospitals across the three nations.

#### 4.2.2 Germany and Japan: significant local government responsibility

In governance and responsibility sharing, both Germany and Japan highlight the role of local governments, though their models differ. German hospitals operate under a dual-track system separating investment from operations. State governments bear capital expenditures for hospital infrastructure and major equipment procurement, using fiscal budgets to ensure ongoing hardware upgrades. Routine operational funding primarily relies on statutory health insurance revenues from medical services, creating a government-funded infrastructure, insurance-funded operations framework. This effectively decouples long-term development investments from short-term operational pressures (38). Japan's public hospital financing responsibilities and functional divisions rely on central-local collaboration. Local governments bear core responsibilities in construction and operation, supporting hospital funding needs through taxation and fiscal subsidies. Local governments in remote and financially weak regions face greater pressure, with their public hospitals highly dependent on central government transfer payments and policy support. Within this framework, Japan implements a medical burden-sharing policy where local governments shoulder significant public healthcare responsibilities, while the central government ensures balanced resource allocation and sustainable medical services through fiscal subsidies and policy guidance.

Since implementing the Plan for Deepening the Reform of the Medical and Health System in 2009, China has assigned local governments a crucial role in public hospital construction and operation, with central government support provided through transfer payments and fiscal subsidies (35). However, disparities in local fiscal capacity have led to significant financial pressures in impoverished regions, resulting in uneven distribution of healthcare resources and compromising the equity and accessibility of medical services. Even with central government support, the quality and accessibility of healthcare services in some resource-deprived areas remain affected, limiting the overall efficiency and fairness of the healthcare system (39). In summary, Japan ensures balanced resource allocation through effective central support and policy subsidies, while local governments bear primary fiscal responsibility. China, however, faces resource distribution disparities due to regional fiscal capacity differences, impacting healthcare service quality and accessibility.

#### 4.2.3 Singapore excels in subsidy design and group management

Since the 1980s, Singapore has implemented systematic reforms in public hospital management, establishing a healthcare system centered on competition, decentralization, responsiveness, and efficiency. The refinement of subsidy design and the systematization of group management are two key factors in the success of its healthcare system. Singapore's healthcare subsidy system is characterized by “tiered pricing,” which ensures equitable access to basic care while controlling government expenditure. Public hospital wards are categorized into five tiers—A, B1, B2+, B2, and C—receiving subsidies of 0%, 20%, 50%, 65%, and 80%, respectively. High-income individuals pay more for premium wards, while low-income groups receive substantial subsidies for basic care, embodying the equitable principle of cross-subsidization. For financing, Singapore has established a multi-tiered protection system anchored by the 3M Plan. In public hospital management, Singapore implemented group-based and corporate reforms, introducing market competition and decentralized governance to enhance healthcare efficiency and quality. Group-based reforms have optimized resource allocation and service coordination. For instance, Singapore Health integrates multiple hospitals, specialist centers, and polyclinics to enable electronic health record sharing, two-way referrals, and centralized procurement. This significantly reduces operational costs while improving service quality (31).

In summary, Singapore's successful experience in subsidy design and group management demonstrates the government's governance wisdom in enhancing system efficiency through market mechanisms while ensuring equitable access to basic healthcare. Its tiered subsidy system controls government expenditure while guaranteeing fair and accessible healthcare services; the group-based and corporate management model improves resource allocation efficiency and service responsiveness through decentralization, competition, and integration.

### 4.3 China's positioning

China's public hospitals are undergoing profound transformation in their positioning and support policies. Since the new round of healthcare reforms, the Chinese government has progressively strengthened its responsibilities, affirming public hospitals' leading role in providing essential healthcare services and offering compensatory measures such as fiscal investments (35). However, the current supportive policy framework still faces multiple challenges: First, insufficient policy coordination leads to fragmentation, with reforms in fiscal investment, medical insurance payments, and personnel compensation failing to fully synchronize, potentially offsetting policy effects. Second, inadequate government funding leaves some hospitals grappling with policy-induced deficits and operational pressures. Third, although a tiered healthcare system has established a preliminary framework, its implementation has exposed issues such as low rates of primary care utilization and inadequate supporting mechanisms (40). Specific details are shown in Figure 1.

## 5 Implications for China from the development trajectories of supportive policies for public hospital development in major global economies

### 5.1 Fiscal and investment mechanism level

Germany employs a dual-track system for hospitals: state governments fund capital expenditures like infrastructure and major equipment through fiscal budgets, ensuring continuous hardware upgrades; routine operational costs are primarily covered by statutory health insurance revenues (38). This creates a government-funded infrastructure, insurance-funded operations framework, effectively separating long-term development investments from short-term operational pressures. Japan's public hospital construction and development funding is jointly borne by central and local governments alongside the hospitals themselves. Central government transfers support underdeveloped regions, reflecting an equity-oriented approach. Its universal social health insurance system makes operations dependent on insurance payments. Additionally, the Japanese government provides public service subsidies to public medical institutions under independent administrative agencies, broadening their financing channels. This alleviates the conflict between public service provision and capital appreciation, helping them adapt to market competition.

Drawing from German and Japanese experiences, strengthening China's central-local fiscal sharing mechanism can be approached through two avenues: First, clarify the fiscal responsibilities and expenditure obligations between central and local governments. Further define the central government's investment responsibilities in areas such as regional medical center construction and major public health projects, while establishing local governments' primary responsibility for ensuring basic infrastructure development and resolving historical debts of public hospitals within their jurisdictions, thereby building a stable, long-term investment mechanism. Second, implement a dual-track budget management model. Divide public hospital budgets into development budgets and operational budgets. The former, funded by fiscal guarantees, supports infrastructure construction and equipment upgrades aligned with regional health plans. The latter is primarily financed through mechanisms like medical insurance payments linked to service volume and performance, incentivizing hospitals to enhance operational efficiency and service quality.

### 5.2 Payment and pricing mechanisms

The UK NHS's DRG payment system promotes hospital cost control and quality improvement while ensuring healthcare equity through nationally standardized criteria. This offers valuable insights for China's deepening medical insurance payment reform. China should refine the linkage mechanism between medical insurance payments and hospital performance evaluations, tightly tying payment standards to performance indicators such as service quality, operational efficiency, and patient satisfaction. This approach incentivizes hospitals to enhance service quality and efficiency, enabling value-based resource allocation and improving the utilization efficiency of medical insurance funds. The UK operates its universally covered NHS system with a supporting outcomes framework developed by the Department of Health and Social Care. This framework encompasses service quality, clinical environment, and patient experience to measure public hospital performance and improve health outcomes. Concurrently, the UK innovatively established a quality-focused payment mechanism where hospital total revenue comprises basic service fees (approximately 70%) and variable performance payments linked to healthcare quality outcomes (approximately 30%). This mechanism uses economic incentives to drive service quality improvements while rationally controlling overall healthcare costs through measures like optimizing clinical pathways. Germany, the birthplace of social health insurance, employs a DRG payment system covering most inpatient services, supplemented by bed-day payments and other complementary methods to form a diversified payment model (41). Additionally, healthcare service prices are negotiated between hospital associations and health insurance associations.

Drawing on British and German experiences, advancing DRG/DIP payment reforms and establishing a value-based healthcare pricing system can proceed through two key approaches: First, deepen DRG/DIP payment method reforms. Building on comprehensive coverage, continuously optimize grouping schemes and weighting parameters. Establish a value evaluation system for disease groups and conditions, incorporating technical complexity, risk level, and resource intensity as core pricing factors to reflect the value of medical professionals' technical labor. Second, establish an integrated mechanism linking price formation, payment methods, and performance evaluation. Explore a dynamic medical service price adjustment mechanism led by medical insurance authorities with participation from healthcare institutions and experts. Link price adjustments to DRG/DIP payment standards and performance metrics such as medical quality, patient safety, and cost control, forming a closed-loop management system.

### 5.3 Governance and legal entity reform

Japan implemented the National Medical Institution System under independent administrative corporations, transforming public hospitals into autonomous, self-financing legal entities. Singapore restructured public hospitals into vertically integrated healthcare groups, each operating as a company with an independent board. Within budgetary frameworks, these groups make autonomous decisions and coordinate internal resources to deliver coordinated services spanning primary care to tertiary hospitals. Drawing from Japan's hospital incorporation and Singapore's public hospital group governance models, enhance hospital autonomy and accountability. First, progressively advance the group-based and incorporated governance of public hospitals. Encourage qualified public hospitals within regions to form tightly integrated healthcare groups under unified management. Fully implement operational autonomy for public hospitals in personnel management, internal compensation distribution, and budget execution. Second, establish a corporate governance structure centered on the board of directors. Create robust hospital boards comprising government departments, community representatives, experts, and employee delegates to oversee strategic decisions and supervise hospital directors. Clearly delineate the authority and responsibilities of the board, hospital director, and Party committee to establish a modern governance system featuring mutual checks and balances among decision-making, execution, and oversight.

### 5.4 Tiered healthcare system with integration

Singapore has established a clearly structured tiered healthcare system through systematic policy guidance and institutional design. This system employs a dual-tier service network architecture: the upper tier comprises general hospitals and specialist hospitals handling critical and emergency care, while the lower tier consists of primary care clinics spread across communities. Through defined functional divisions and effective referral mechanisms, it achieves optimized allocation of medical resources and standardized management of patient care pathways. At the policy level, patient flow is effectively guided by significantly increasing the out-of-pocket costs for patients bypassing primary care to access specialist outpatient services directly, while providing subsidies and priority appointment access to those referred through community clinics (32). Drawing from Singapore's experience, Singapore has strengthened incentives for primary care first-visit and referral systems. First, it enhances economic and institutional levers for primary care first-visit. Further widen the gap between primary care institutions and tertiary hospitals in terms of medical insurance reimbursement rates and deductible thresholds to create a strong economic incentive. Simultaneously, establish convenient and seamless electronic referral channels. Second, establish an integrated service model combining medical care and prevention. Drawing on Singapore's healthcare model, promote the transformation of China's community health service centers into health management centers that provide basic medical care, public health services, and health management, positioning them as the first point of entry in the regional healthcare system.

### 5.5 Performance evaluation and incentive framework

The value-based care performance frameworks developed in countries like the UK and the US. From the UK's Quality and Outcomes Framework (QOF), as societal contexts evolve and the value of healthcare services expands, payment designs increasingly aim to achieve organic coordination among patients, general practitioners, and the NHS. The dimensions of payment value are also becoming increasingly diversified. The U.S. CMS has vigorously promoted value-based payment models within Medicare and Medicaid, building upon the core of performance-based payments. This has led to the development of the Value-Based Purchasing Program (VBP) (42). Introducing multidimensional performance metrics linked to healthcare provider incentives. First, establish a multidimensional performance metric system tailored to China's national context. This system should balance multiple dimensions, including quality, safety, efficiency, cost control, and patient satisfaction. For example, incorporate metrics reflecting quality and efficiency such as the proportion of same-day surgeries, antibiotic usage intensity, and the percentage of minimally invasive procedures among discharged patients. Second, achieve precise linkage between performance evaluation and compensation distribution. Further refine the compensation structure for medical personnel by establishing a “base salary + performance bonus” model. Performance bonuses should primarily derive from savings generated by improving service quality and controlling unreasonable costs, rather than solely from business revenue. This approach will eliminate the entrenched practices of subsidizing medical care through drug sales and subsidizing medical care through diagnostic testing.

### 5.6 In the realm of digital and smart healthcare

Germany mandates electronic health records (EHR) through legislation and standardizes telemedicine applications and reimbursement criteria, enhancing healthcare data interoperability and service accessibility. Singapore has established a National Electronic Health Record (NEHR) system, integrating GP workstations with the national EHR. By consolidating primary care, healthcare insurance, pharmaceutical management, and public health data, it enables rational resource allocation and precise disease surveillance. Advancing Digital and Smart Healthcare. First, enhance the standardization and interoperability of healthcare big data. Break down “information silos” between medical institutions by unifying data standards and interfaces at the national level, facilitating secure sharing and operational coordination of electronic medical records and health archives across healthcare facilities of different tiers. Second, vigorously develop Internet Plus Healthcare and artificial intelligence applications. Increasingly incorporate telemedicine and smart hospital development into the performance evaluation system for public hospitals. Encourage the use of AI technology to assist clinical diagnosis, hospital management, and drug research and development, enhancing the precision of medical services and operational efficiency to provide the public with more convenient and efficient smart healthcare services.

### 5.7 Regionally differentiated strategies for China

China's public hospital reform needs to account for substantial regional disparities in fiscal capacity, demographic conditions, and healthcare development levels. Uniform national policy approaches may therefore produce uneven outcomes across regions. In economically developed eastern provinces, strong local government revenues have enabled rapid expansion of hospital infrastructure and high-end medical services. While this has improved service availability, several studies note growing concerns over service duplication, inefficient resource utilization, and rising operational costs in some urban areas (43). Policy priorities in these regions should focus on moderating expansion, strengthening regional service coordination, and improving efficiency through hospital integration and performance-based management. By contrast, many hospitals in central and western regions face persistent constraints related to limited fiscal capacity, historical debt burdens, and insufficient investment in infrastructure and workforce development (44). These challenges restrict their ability to upgrade facilities, retain qualified medical personnel, and expand essential service capacity. For these regions, stronger central government transfers, targeted capital investment programs, and inter-regional cooperation mechanisms are needed to support basic service provision and reduce structural inequalities in healthcare access. Accordingly, future public hospital reforms in China should adopt regionally differentiated strategies that align policy instruments with local fiscal conditions and development stages, rather than relying on uniform national implementation models.

## 6 Conclusion

This paper systematically examines the policy trajectories supporting public hospital development in five representative economies, namely the United States, the United Kingdom, Germany, Japan, and Singapore, revealing the evolutionary logic and practical outcomes of policy tools under diverse healthcare models and governance structures. Research indicates that despite differences in institutional contexts, policy evolution across these countries consistently reflects a sustained balance between ensuring basic healthcare provision, enhancing service system efficiency, and addressing structural challenges. International experience suggests that the effective operation of public hospitals requires both stable fiscal investment or robust health insurance systems as a foundation, and coordinated reforms in governance mechanisms, payment methods, and system integration. Practices in areas such as legal person status, group-based management, tiered healthcare system, and digital transformation offer valuable references for China to optimize public hospital governance structures and build integrated service systems. Future research could further integrate China's regional disparities and reform practices to conduct more targeted policy impact assessments on key issues like payment method reforms, defining hospital autonomy, and implementing tiered diagnosis and treatment. This would provide more actionable pathways for constructing a high-quality public hospital system tailored to China's national conditions.

## Data Availability

The original contributions presented in the study are included in the article/supplementary material, further inquiries can be directed to the corresponding authors.
